# Antiviral Defenses in Plants through Genome Editing

**DOI:** 10.3389/fmicb.2017.00047

**Published:** 2017-01-23

**Authors:** Gustavo Romay, Claude Bragard

**Affiliations:** Applied Microbiology – Phytopathology, Earth and Life Institute, Université catholique de LouvainLouvain-la-Neuve, Belgium

**Keywords:** antiviral defense, CRISPR/CAS9, genome editing technologies, plant viruses, TALEN, ZFN

## Abstract

Plant–virus interactions based-studies have contributed to increase our understanding on plant resistance mechanisms, providing new tools for crop improvement. In the last two decades, RNA interference, a post-transcriptional gene silencing approach, has been used to induce antiviral defenses in plants with the help of genetic engineering technologies. More recently, the new genome editing systems (GES) are revolutionizing the scope of tools available to confer virus resistance in plants. The most explored GES are zinc finger nucleases, transcription activator-like effector nucleases, and clustered regularly interspaced short palindromic repeats/Cas9 endonuclease. GES are engineered to target and introduce mutations, which can be deleterious, via double-strand breaks at specific DNA sequences by the error-prone non-homologous recombination end-joining pathway. Although GES have been engineered to target DNA, recent discoveries of GES targeting ssRNA molecules, including virus genomes, pave the way for further studies programming plant defense against RNA viruses. Most of plant virus species have an RNA genome and at least 784 species have positive ssRNA. Here, we provide a summary of the latest progress in plant antiviral defenses mediated by GES. In addition, we also discuss briefly the GES perspectives in light of the rebooted debate on genetic modified organisms (GMOs) and the current regulatory frame for agricultural products involving the use of such engineering technologies.

## Introduction

Viruses are well-known to be one of the major concerns for agricultural production and food security throughout the world. It is estimated that viral agents are responsible for the half of emerging diseases reported in plants (Anderson et al., 2004). The control of plant viruses is often dependent on the use of pesticides; however, such strategy has many adverse environmental effects (Bragard et al., 2013). In many plant virus-related outbreaks, the disease management is difficult to accomplish due to the variability of factors affecting the development of the disease, such as local climate conditions, plant aging, crop varieties, vector transmission efficiency and severity of viral strains. Unlike other pathogens (i.e., fungi and bacteria), plant viruses cannot be controlled chemically and a combination of cultural practices, biosecurity measures, organism-vector management and plant genetic resistance is needed to deal with the disease (Nicaise, 2014).

The use of viral resistance factors from plants is considered one of the most important alternatives to face virus infections (Hull, 2014; Ziebell, 2016). The pioneer works on resistance to *Tobacco mosaic virus* in *Nicotiana glutinosa* led to the initial understandings on plant viral immune responses and the introgression of resistance genes from wild to cultivated plants (Mandadi and Scholthof, 2013). Over the past decades, virus resistance genes have been used to improve the most of cultivated plants and many cultivars are commercially available (Gómez et al., 2009). The main drawbacks of approaches using resistance genes are the considerable time and cost to develop a durable resistant crop variety (Kang et al., 2005).

Plants, like other eukaryotes, are able to deploy an alternative strategy to face viruses: RNA interference (RNAi). The RNAi is a biological mechanism whereby small RNA molecules, such as small interfering RNA (siRNA) or microRNA (miRNA), can regulate gene functions via post-transcriptional gene silencing. A critical breakthrough was the demonstration that double-strand RNA (dsRNA) molecules trigger the RNAi pathway to regulate gene expression in *Caenorhabditis elegans* (Fire et al., 1998). This model was subsequently tested and confirmed in many other organisms. Nowadays, it is known that these dsRNAs are targeted and cleaved by the endoribonuclease Dicer producing 21 to 25-nucleotide small RNAs (siRNA or miRNA) which are bound to an Argonaute protein into the RNA-induced silencing complex (RISC) (Wilson and Doudna, 2013). The RISC is guided by the Argonaute-bound strand to a single strand RNA, perfectly complementary to the dsRNA, which is degraded or translationally inhibited (Bartel, 2009; Wilson and Doudna, 2013). In plants, dsRNA molecules from either RNA or DNA viruses may be produced and afterward processed by the host RNAi machinery to induce an antiviral response (Szittya and Burgyán, 2013). Although the RNAi triggering molecule (i.e., dsRNA) had not been discovered in the 1980s, it was known that the inhibition of gene expression could be generated by expression of antisense RNA in plant cells (Ecker and Davis, 1986). The application of this strategy to induce pathogen resistance, involving the pathogen genome itself, was called parasite-derived resistance (Sanford and Johnston, 1985), and currently referred as pathogen derive resistance (PDR). Since then, this approach has been used to derive viral resistance through transgenic expression of virus genes in plants and, in some cases, with commercial applications (Baulcombe, 1996; Simón-Mateo and García, 2011; Younis et al., 2014). However, most viruses have developed silencing suppressor mechanisms to counteract the RNAi-mediated defense of plants. Hence, an RNAi-mediated resistance in transgenic plants could be overcome by the targeted virus after inoculation with a non-target virus possessing a silencing suppressing gene (Simón-Mateo and García, 2011). Besides, RNAi technology is based on knockdown gene function(s), which can be incomplete, varies between different experiments and have unpredictable off-target effects (Gaj et al., 2013). Therefore, other approaches involving stable gene modification have been gaining attention over the last decade due to their versatility to manipulate any gene from any organism (Gaj et al., 2013; Boettcher and McManus, 2015). These approaches are referred as genome editing systems (GES). In this review, we provide insights about the latest progress on the different technologies based on GES used to control plant viruses and its perspective for a broad application in crop improvement.

## Mechanisms of Genome Editng Systems

Prior to GES development, the genetic engineering of virus resistant plants has been mainly undertaken using viral sequences, which are introduced in the genome of the susceptible plant through genetic transformation methods (Saharan et al., 2016). At least 25 viruses have been used to develop virus resistant plants by inserting the viral sequence itself (Wani and Sanghera, 2010; Saharan et al., 2016). Although several techniques for genetic transformation of plants are available, the most popular techniques are *Agrobacterium* infection and ballistic bombardment (Ye, 2015). *Agrobacterium-*mediated transformation is based on the transfer and insertion of a given DNA sequence into a plant genome by plasmids Ti (tumor-induced) or Ri (rhizogenic) from the bacterium (Gelvin, 2003). Genetic transformation trough ballistic bombardment is based on delivery of DNA-coated metal particles accelerated with a biolistic device to introduce the DNA into the target cells or tissues (Kikkert et al., 2005). Both methods have been used to develop antiviral strategies focused on PDR approach. When the complete sequence of a virus gene is inserted into the host plant genome, to interfere with the life cycle of the target virus, the PDR approach is referred as viral protein mediated resistance (Saharan et al., 2016). Within such approach the major viral proteins used are the coat protein, replicase protein, movement protein, and replication-associated protein (Prins et al., 2008). As mentioned above, the PDR approach have been also applied using small sequences from the viral genome to activate the RNAi mechanism of the host plant, via post-transcriptional gene silencing (Simón-Mateo and García, 2011).

**FIGURE 1:**
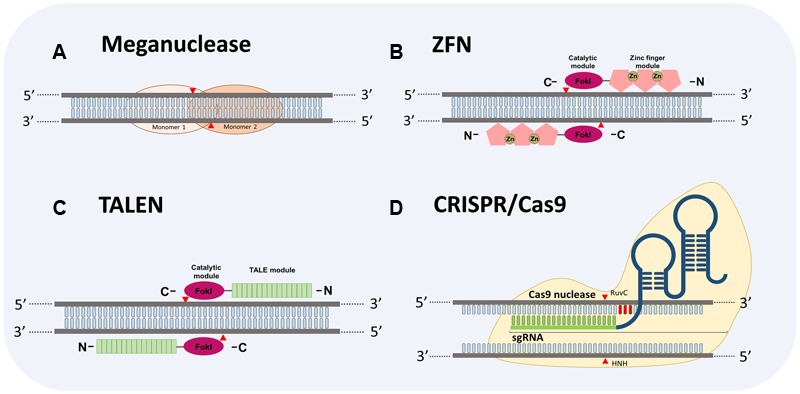
**Schematic diagram depicting four Genome Editing Systems (GES) to target DNA. (A)** Homodimers structure of a meganuclease system. **(B)** Zinc finger nuclease (ZFN) showing two monomers bound to DNA. The ZFN contains a catalytic FokI domain (ellipse in pink) and a zinc finger DNA-binding domain (DBD) (pentagons in rose). **(C)** Transcription activator-like effector nuclease (TALEN) showing two monomers bound to DNA. Like ZFN, TALEN comprises a catalytic FokI domain (ellipse in pink). Light green rectangles represent the DNA bind domain containing the repeat variable di-residue (RVD) arrays of amino acids to recognize DNA specific sequences. **(D)** Clustered regularly interspaced short palindromic repeats (CRISPR) CRISPR-associated protein 9 (Cas9) (CRISPR/Cas9). Typically CRISPR/Cas9 system comprises a Cas9 protein (depicted in light gold) with two nuclease domains, referred as HuvC and HNH, and a chimeric single guide RNA (sgRNA). The sgRNA consists of a CRISPR RNA (crRNA, 21 nucleotides in light green) to direct the Cas9 protein to the complementary sequences of the DNA target and a *trans*-activating crRNA (RNA sequence represented in dark blue) involved in the processing of pre-crRNA into a mature crRNA. Arrowheads in red indicate cleave sites to each GES.

Genome editing systems approaches against plant viruses have been mainly developed using *Agrobacterium* infection to introduce such systems into the plant cells for stable or unstable transformation. The applications of GES are based on the use of sequence-specific nucleases, which lead to DNA modifications by double-strand breaks (DSBs) in a targeted gene (Gaj et al., 2013; Voytas, 2013). After the DSBs DNA is repaired by two different mechanisms: (i) the non-homologous end-joining (NHEJ), in which the ends of the broken DNA are re-joined without use of a repair template, and (ii) the homologous recombination (HR) whereby two homologous DNA molecules exchange nucleotide sequences (Wyman and Kanaar, 2006). Thus, DNA modifications mediated by sequence-specific nucleases are possible in a particular genomic location (Voytas, 2013). Four major platforms for GES have been developed using sequence-specific nucleases (**Figure 1**), meganucleases, zinc finger nucleases (ZFNs), transcription activator like effector nucleases (TALENs) and more recently the clustered regularly interspaced short palindromic repeats (CRISPR) along with the CRISPR-associated protein 9 (Cas9) (CRISPR/Cas9).

Meganucleases or homing endonucleases are encoded by introns and inteins that recognize DNA sequences between 12 and 42-base-pair (bp) in length, unlike restriction enzymes (Jurica and Stoddard, 1999). The meganucleases are characterized by a high specificity, even though they could tolerate single mutations in the targeted sequence (Silva et al., 2011). Due to the high specificity of meganucleases, the repertoire of targetable sequences is very limited (Pâques and Duchateau, 2007). In specific cases their utility relies on the previous insertion of the recognition site in the targeted genome to undertake a high-efficiency recombination (Carroll, 2011). To overcome this drawback, residues-specific mutations in engineering meganucleases are introduced to alter their DNA recognition sites allowing to increase the use of these proteins in gene targeting experiments, however, the production of customized meganucleases still remains too complex (Silva et al., 2011).

The second GES are the ZFNs, which are chimeric proteins created by fusing the DNA-binding domain (DBD) of a zinc-finger protein with the DNA cleavage domain of the *Fok*I restriction enzyme (Urnov et al., 2010). *Fok*I works as a dimer and its catalytic domain cleaves the DNA sequence outside of the recognition site (Bitinaite et al., 1998). A ZFN is engineered with two monomers separated by a spacer sequence of 5–7 bp wherein the catalytic domains of the chimeric proteins cleave each DNA brand to produce the DSB (Christian et al., 2010). An effective ZFN should contain more than three zinc-finger domains in each DNA-binding module to increase specific DNA recognition (Gaj et al., 2013).

Transcription activator-like effector nucleases is the third GES. This system is a fusion of a transcription activator-like effector (TALE) and the non-specific cleavage domain of the enzyme *Fok*I (Cermak et al., 2011; Mahfouz et al., 2011; Pesce et al., 2015). TALEs are proteins encoded by phytopathogenic bacteria *Xanthomonas* spp. and delivered into the plant host cells to promote pathogen growth through manipulation of plant processes (Boch and Bonas, 2010; Schornack et al., 2013). Once TALEs are injected into the cells, they translocate to the nucleus, bind to their DNA targets and mimic host transcription factors to reprogram host gene expression (Mahfouz et al., 2011). TALE proteins are composed by an N-terminal secretion and translocation domain, a central DBD and a C-terminal transcription activation domain carrying nuclear localization signal (Schornack et al., 2013). The DNA-binding specificities of these proteins were solved by Boch et al. (2009) who showed that the DBD is an array of tandem repeat units consisting of 34 amino acids with two hypervariable amino acids at the position 12 and 13 that constitute a repeat variable diresidue (RVD). A specific amino acid arrangement in the RVD region determine a specific nucleotide recognition in the DNA target (Boch et al., 2009). Thus, this characteristic of TALEs, along with the previous knowledge on biotechnology applications of *Fok*I enzyme, have allowed the TALEN system’s design.

The fourth and most recent GES is CRISPR/Cas9, which is an RNA-guided nuclease technology. This genome editing approach is based on the CRISPR/Cas system found in most archaea and many bacteria that confers immunity against foreign DNA elements such as viruses and plasmids (Barrangou and Marraffini, 2014; Makarova et al., 2015). Barrangou et al. (2007) demonstrated that after viral challenge on several strains of *Streptococcus thermophilus* the bacteria are able to generate virus-resistant mutants through integration of viral genome sequences into the CRISPR loci in association with *cas* genes expression. Among CRISPR/Cas systems, the CRISPR/Cas9 from *S. pyogenes* is the most studied model (Dominguez et al., 2016). Two elements are essential to engineer a CRISPR/Cas9 system: (i) the *cas*9 protein containing two nuclease domains (RuvC and HNH) that cleave both strands of the DNA target leading to DSBs and site mutations, and (ii) a guide RNA (gRNA) whose role is direct *cas*9 protein to the DNA target. A gRNA is composed by two different RNA molecules: a CRISPR RNA (crRNA), which contains complementary sequences to the DNA target, and a *trans*-activating crRNA (tracrRNA) involved in processing of precursor crRNA molecules to a mature crRNA (Brouns et al., 2008).

**FIGURE 2:**
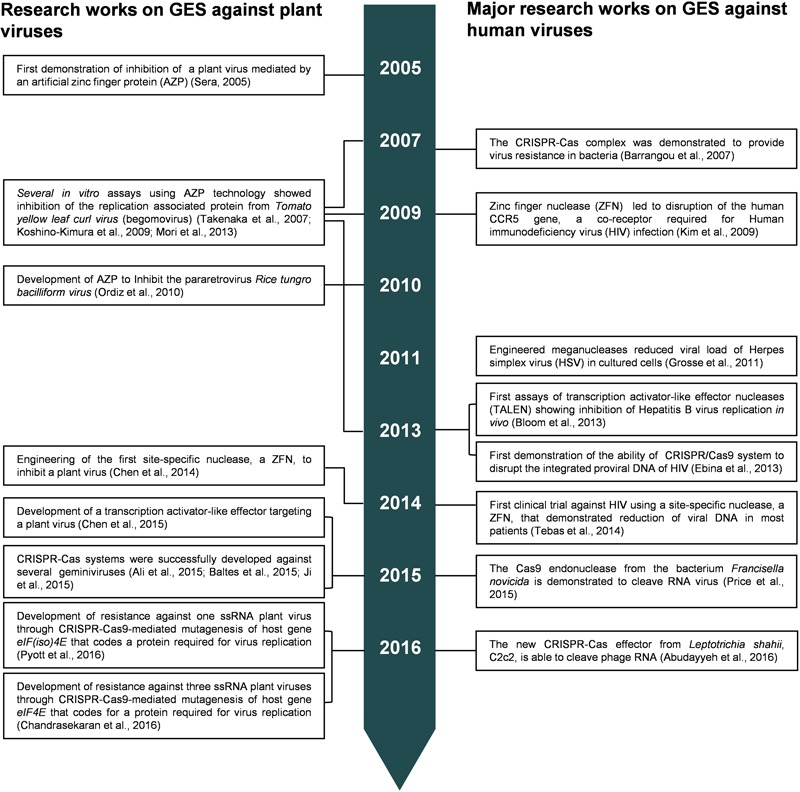
**Highlights on developments of genome editing technologies against viruses**.

A milestone in the development of CRISPR/Cas as a biotechnological tool was the engineering of a chimeric RNA containing the crRNA and tracrRNA in a single guide RNA (sgRNA), which was also able to direct Cas9 to the DNA target (Jinek et al., 2012). After such a finding, CRISPR-Cas9 technology became the most popular GES. The specificity of the CRISPR/Cas9-mediated DNA cleavage also relies on recognition of a trinucleotide sequence of DNA target referred as protospacer adjacent motif (PAM) (Sternberg et al., 2014). As showed in **Figure 2**, CRISPR-Cas technology is rapidly advancing and expanding its potential application not only targeting DNA molecules, like the precedent genome editing technologies, but also RNA molecules including RNA viruses.

As mentioned above, GES such as ZFN, TALEN, or CRISPR-Cas9 are provided of DNA-binding and catalytic domains. Meanwhile, TALE and artificial zinc finger protein (AZP) have DBDs and lack of catalytic domains. TALE and AZP can be engineered to prevent viral multiplication by blocking specific DNA sites in the viral genomes, which are essential for DNA-binding proteins of the virus and subsequent interactions with the replication machinery of host cell.

## Genome Targeting Technologies and GES Against Plant Viruses

In **Table 1** are summarized the several genome targeting technologies that have been explored to provide potential control against plant viruses. It is important to note that the most evaluated species belong to the families *Geminiviridae* and *Potyviridae*. According to the latest report of the International Committee on Taxonomy of Viruses (ICTV), *Geminiviridae* and *Potyviridae* families represent the two largest groups of plant viruses containing 326 and 195 species, respectively (ICTV, 2015). Hence the use of these technologies to enhance plant resistance against a large number of viruses might increase significantly in the near future.

**Table 1:** Genome targeting technologies developed to confer viral resistance in plants.

Genome targeting platform	Virus	Genus	Family	DNA targeted	Reference
CRISPR/Cas9	*Merremia mosaic virus, Cotton leaf curl Kokhran virus, Tomato yellow leaf curl virus* (TYLCV)	*Begomovirus*	*Geminiviridae*	Viral	Ali et al., 2015, 2016
	*Beet curly top virus* (BCTV); *Beet severe curly top virus* (BSCTV)	*Curtovirus*		Viral	Ali et al., 2015; Ji et al., 2015
	*Bean yellow dwarf virus*	*Mastrevirus*		Viral	Baltes et al., 2015
	*Cucumber vein yellowing virus*	*Ipomovirus*	*Potyviridae*	Host	Chandrasekaran et al., 2016
	*Zucchini yellow mosaic virus, Papaya ringspot virus, Turnip mosaic virus*	*Potyvirus*		Host	Chandrasekaran et al., 2016; Pyott et al., 2016
TALE	*Tomato yellow leaf curl China virus* (TYLCCNV), *Tobacco curly shoot virus* (TbCSV), *Tomato leaf curl Yunnan virus*	*Begomovirus*	*Geminiviridae*	Viral	Cheng et al., 2015
ZFN	TYLCCNV, TbCSV	*Begomovirus*	*Geminiviridae*	Viral	Chen et al., 2014
AZP	BSCTV, TYLCV	*Begomovirus*	*Geminiviridae*	Viral	Sera, 2005; Takenaka et al., 2007; Koshino-Kimura et al., 2009; Mori et al., 2013
	*Rice tungro bacilliform virus*	*Tungrovirus*	*Caulimoviridae*	Viral	Ordiz et al., 2010

### Antiviral Resistance in Plants Based on Zinc Finger Technology

The first efforts to introduce a viral inhibition factor in plants, through zinc finger technology, were carried out in the mid-2000s. Sera (2005) developed an AZP *in planta* targeting a 19-bp fragment in the intergenic region (IR) of *Beet severe curly top virus* (BSCTV, genus *Curtovirus*). BSCTV, like all members of family *Geminiviridae*, has an IR containing a binding site recognized by the replication initiator protein (Rep) to initiate viral replication (Rizvi et al., 2015). BSCTV replication was reduced in transgenic *Arabidopsis thaliana* plants carrying the AZP that was efficient to block the binding site of the BSCTV Rep (Sera, 2005). *In vitro* assays, using AZP technology, have also been performed to predict inhibition of *Tomato yellow leaf curl virus* (TYLCV, genus *Begomovirus*) by blocking the Rep binding site of TYLCV (Takenaka et al., 2007; Koshino-Kimura et al., 2009; Mori et al., 2013); however, its efficient application *in planta* remains to be confirmed. Zinc finger technology was also applied to reduce replication of *Rice tungro bacilliform virus* (RTBV, genus *Tungrovirus*) in transgenic *A. thaliana* plants carrying an AZP which was able to recognize and block promoter sequences of RTBV (Ordiz et al., 2010). AZP for antiviral applications have been designed to bind and block specific DNA sites that are crucial for DNA-binding proteins of viruses (Sera, 2005; Koshino-Kimura et al., 2009). Unlike AZP, the ZFN technology, for antiviral applications, involves not only the DBD but also the DNA catalytic domains of *Fok*I restriction enzyme to introduce deleterious mutations in the viral genome, as previously explained. More recently, ZFN strategy against *Tomato yellow leaf curl China virus* (TYLCCNV, genus *Begomovirus*) was developed to target the AC1 gene of the virus that codes the Rep protein (Chen et al., 2014). In that study, agroinfiltrations of *N. benthamiana* plants with TYLCCNV infectious clone and antiviral ZFN showed a significant reduction of viral replication as compared with agroinfiltrations of the viral clone alone. Furthermore, the same ZFN developed for TYLCCNV was tested against *Tobacco curly shoot virus* (TbCSV), another begomovirus, showing an inhibition of the replication of the virus and, thus, suggesting a possible resistance strategy to be broadly used against begomoviruses.

### Antiviral Resistance in Plants Based on TALE Technology

Recently Cheng et al. (2015) developed a TALE platform to evaluate a broad-spectrum resistance against begomoviruses. In such study, two conserved 12-nucleotide motifs among begomoviruses (into the IR and the AC1 gene, respectively) were used to engineer TALEs and, afterward, were challenged with three begomoviruses: TbCSV, *Tomato leaf curl Yunnan virus* (TLCYnV) and *Tomato yellow leaf curl China virus* (TYLCCNV). Transgenic plants of *N. benthamiana* carrying the TALEs displayed resistance to TbCSV and TYLCCNV, while the resistance to TLCYnV was partial. Although a broad-spectrum approach for resistance to a virus group is desirable, it seems difficult to predict a unique TALE system to control a large group of virus like geminiviruses. TALE technologies including nuclease domains (TALEN) have not been still reported against plant viruses. For human viruses, the potential antiviral applications of TALEN have been explored for several viruses such as Human immunodeficiency virus (HIV), Hepatitis B virus (HBV), Hepatitis C virus (HCV), however, the generic challenges for gene therapy (e.g., adequate specificity, viral escape, efficient delivery to virus infected tissues, or limited immune host responses) remain to be solved (Bloom et al., 2015).

### Antiviral Resistance in Plants Based on CRISPR-Cas Technology

Since the advent of CRISPR-Cas system as a biotechnology tool for genome editing, many labs working with eukaryotic viruses have directed their interest on this technology due to its affordability, simplicity and efficiency as compared with precedent GES like ZFN or TALEN. Thus, many efforts are being undertaken to shed light on the potential application of CRISPR-Cas9 to control human viruses such as HIV, HBV, Human papillomavirus, Epstein-Barr and plant viruses (Price et al., 2016).

As showed in **Table 1**, four and six viruses in the families *Potyviridae* and *Geminiviridae*, respectively, have been used for developing antiviral defenses in plants, using CRISPR-Cas systems (Ali et al., 2015, 2016; Baltes et al., 2015; Chandrasekaran et al., 2016; Pyott et al., 2016). Interestingly, independent studies evaluating geminivirus resistance showed that the most promising sgRNAs were those targeting the IR of these viruses (Ali et al., 2015, 2016; Baltes et al., 2015). The IR of geminiviruses contain a stem-loop structure in which an invariant nonanucleotide motif is involved in the viral replication (Hanley-Bowdoin et al., 2000). Baltes et al. (2015) observed that sgRNAs designed near to the stem-loop structure of *Bean yellow dwarf virus* were less efficient to generate insertions/deletions (indels), suggesting a possible interference of the secondary structure on the sgRNA-Cas9 cleavage. Despite that, viral load was reduced probably by blocking of the Rep binding site (Baltes et al., 2015). Furthermore, Ali et al. (2015) engineered a sgRNA-Cas9 that was efficient to target the IR of TYLCV and also that of other geminiviruses like *Beet curly top virus* and *Merremia mosaic virus*. More recently, sgRNA-Cas9 targeting the IRs and the coat protein genes of the TYLCV and *Cotton leaf curl Kokhran virus* showed that CRISPR-Cas9 directed to coding sequences can generate viral variants which are able to replicate and spread in the plants, while CRISPR-Cas9 directed to non-coding intergenic sequences produced viral interference and a low recovery of mutated viral variants (Ali et al., 2016). One of the most interesting feature of CRISPR-Cas9 system is its flexibility to assemble multiple gRNA modules for targeting several genes simultaneously (Xing et al., 2014; Xie et al., 2015). Baltes et al. (2015) showed that a CRISPR-Cas9 system containing two gRNA modules targeting the same viral genome was more effective to reduce the infection than their relative gRNA delivered in separated constructs.

Given that GES target DNA sequences, these technologies seem to be mainly suitable for plant DNA viruses. However, an interesting CRISPR-Cas9 approach has been successfully used to develop resistant plants against RNA viruses, demonstrating that CRISPR-Cas9 system is a promising and powerful tool to be considered in the near future for crop improvement programs. Two recent studies showed the CRISPR-Cas9-mediated disruption of plant genes encoding eukaryotic translation initiation factors in cucumber and *A. thaliana* (Chandrasekaran et al., 2016; Pyott et al., 2016, respectively). Interactions between eukaryotic translation initiation factors 4E (eIF4E) or its isoform eIF(iso)4E and the viral genome-linked protein (VPg) of potyviruses are required for the virus infection (Robaglia and Caranta, 2006). Natural resistance to potyviruses are generally associated with mutations of host eIF4E or eIF(iso)4E that hamper their interaction with the VPg protein (Estevan et al., 2014; Sanfaçon, 2015). Chandrasekaran et al. (2016) used CRISPR-Cas9 systems to mutate the *eIF4E* gene in cucumber plants conferring resistance to the ipomovirus *Cucumber vein yellowing virus*, and to the potyviruses *Zucchini yellow mosaic virus* and *Papaya ring spot virus*. Similarly, Pyott et al. (2016) developed another CRISPR-Cas9 construct to introduce site specific-mutations in the *eIF(iso)4E* gene of *A. thaliana*, which were efficient to confer resistance to the potyvirus *Turnip mosaic virus* (TuMV) In the light of these studies, CRISPR-Cas9 systems could be developed to target host genes coding for other members in the family of plant translation factors such as eIF4G, eIF(iso)GE, eIF4A-like helicases, eIF3, eEF1A, and eEF1B that are also identified to interplay with protein and viral RNAs (Nicaise, 2014; Sanfaçon, 2015).

## The Plant Virus Range that GES Can Target

Genome editing systems are known to bind to double-stranded DNA (dsDNA) and subsequently introduce a DSB in a sequence-specific manner. Hence, the first studies using genome targeting or genome editing platforms for plant viruses aimed to control DNA viruses (**Table 1**). Although the members of the plant virus families *Geminiviridae* and *Nanoviridae* are composed of single-stranded DNA (ssDNA), they also contain replicative intermediate forms of dsDNA which can be targeted by GES. The other plant virus family possessing DNA genomes is *Caulimoviridae*. Unlike geminiviruses and nanoviruses, the caulimoviruses have a dsDNA genome. Currently, there are 432 virus species that belong to the DNA virus families *Geminiviridae, Nanoviridae*, and *Caulimoviridae* (ICTV, 2015). Others viruses in the families *Metaviridae* and *Pseudoviridae* that infect plants could be directly targeted by GES. Metaviruses and pseudoviruses are reverse transcribing RNA viruses. Although their genomes are single-stranded RNA (ssRNA), they possess replicative dsDNA forms (Boeke et al., 2012; Eickbush et al., 2012), being good candidates to be targeted by GES. In fact, the reverse transcribing RNA virus HIV has already been subjected to GES by targeting its replicative DNA forms known as provirus (Price et al., 2016).

The majority of plant viruses have RNA genomes. More specifically, 836 out of 1268 species recognized by the ICTV are RNA viruses (ICTV, 2015). Although all GES are able to bind DNA molecules, it was shown that Cas9 protein is able to bind and cleave ssRNA when using specially designed PAM-presenting oligonucleotides (O’Connell et al., 2014). Therefore, the development of an RNA-targeting CRISPR/Cas9 complex offers a promising platform to control RNA viruses. Interestingly, two recent discoveries showed that CRISPR-Cas system can directly interfere with RNA virus infections. In the first study, Price et al. (2015) developed a CRISPR-Cas9 system to target HCV (ssRNA virus) using a new variant of Cas9 endonuclease, called FnCas9, capable to cleave ssRNA molecules. FnCas9 is a Cas protein from the bacterium *Francisella novicida*, which is able to repress a endogenous mRNA (Sampson et al., 2013). In the second study, a new Cas protein from the bacterium *Leptotrichia shahii* was characterized and named C2c2. This protein contains two HEPN (Higher Eukaryotic and Prokaryotic nucleotide-binding) RNase domains (Shmakov et al., 2015). In *L. shahii* C2c2 provides resistance to an RNA phage and it was recently demonstrated to be guided by a single crRNA and programmable against ssRNA (Abudayyeh et al., 2016). Taking into account that 784 out of 1268 plant virus species possesses ssRNA, these discoveries could play a prominent role, in the near future, to enhance the current tools for plant virus control.

## Applications of GES Beyond Plant Virus Control in Agriculture

The great success of the RNAi technology was based on the ability to modulate gene expression by post-transcriptional gene silencing mechanisms whereby the role of many genes has been unveiled. GES are not only able to modulate gene expression, but also are useful to introduce nucleotide modifications into the genome of almost every organism. Hence, gene editing technologies seem to have no limits on their applications. Such applications have been extensively reviewed (Gaj et al., 2013; Boettcher and McManus, 2015; Govindan and Ramalingam, 2016; Steinert et al., 2016). In agriculture, the applications of GES have been explored for many purposes in addition to plant virus control. For example, a meganuclease system was developed to confer herbicide tolerance in cotton lines (D’Halluin et al., 2013). Herbicide resistant plants were also generated using zinc finger technologies to target tobacco acetolactate synthase genes that are involved in resistance to imidazolinone and sulphonylurea herbicides (Townsend et al., 2009). TALENs have been successfully used to inhibit the vacuolar invertase gene in potato, which is associated with the accumulation of reducing sugars and high levels of acrylamide, a potential carcinogen, in tubers (Clasen et al., 2016). TALEN and CRISPR/Cas9 systems were demonstrated to target several genes implicated with the phytic acid production in maize seeds (Liang et al., 2014). Phytic acid is a major storage for phosphorus and mineral cations in several crops and it is poorly digested by monogastric animals (Shi et al., 2003). In wheat, TALENs and CRISPR/Cas9 were also used to confer fungi resistant in wheat lines by disrupting of the mildew locus O (*MLO*) gene, which is related with mildew powder susceptibility (Wang et al., 2014). Besides GES targeting plant genomes, CRISPR/Cas9 system was used to inhibit genes encoding polyphenol oxidase that causes browning in common white mushroom (*Agaricus bisporus*) (Waltz, 2016). In general, many successful examples of GES targeting crops have demonstrated the broad spectrum of applications that exhibit these technologies in agriculture beyond plant virus control.

## GES-Mediated Plant Virus Defenses Vis-À-Vis GMO Regulations

The insights on the scope of GES is rapidly advancing throughout the scientific community. For virologists and plant breeders, genome editing technologies is offering an encouraging approach to circumvent labor-intensive and time-consuming methods used in conventional genetic engineering and traditional breeding techniques. Nevertheless, one of the major hurdles of the GES approaches is the public perception in which a product obtained from these technologies is considered as a genetic modified organism (GMO). The GMO definition according to the Cartagena Protocol on Biosafety and the European Union was mainly conceived to distinguish products obtained by conventional plant breeding technologies and those obtained by recombinant DNA technologies (Sprink et al., 2016). Development of transgenic plants implies the transfer of foreign DNA into host cells. For example, several cases of virus-resistant plants have been generated by insertion of partial sequences from viral genomes based on RNAi technology (Simón-Mateo and García, 2011). Unlike, some genome editing approaches allow the insertion of point mutations in the genome of recipient species and afterward it is possible the removal of the encoding sequences of the programmable nucleases. Indeed, Chandrasekaran et al. (2016) introduced mutations in the factor eIF4E of cucumber plants using the CRISPR/Cas9 system, to induce resistance to potyviruses, and subsequently the gene encoding the GES was removed by breeding, leading to transgene-free generations. Likewise, Pyott et al. (2016) generated TuMV resistant plants of *A. thaliana* lacking the CRISPR/Cas9 system after two generations. Insertion of point mutations are also feasible using mutagenic agents. For example, using the technique TILLING (targeting induced local lesions in genomes) point mutations in tomato eIF4 were induced by ethyl methanesulfonate to confer immunity to two potyviruses (Piron et al., 2010). Besides this, crop improvement derivate by mutagenic agents are not considered as GMO due to the lack of foreign DNA. Thus, some genome editing approaches are considered to generate non-transgenic plants (plant without foreign DNA). However, the controversy whether the products generated by these type of technologies should be considered GMOs or not still remains (Sprink et al., 2016). In another example of transgene-free plants modified by GES, a preassembled CRISPR/Cas9 complex was successfully delivered to induce mutations into protoplasts of *A. thaliana*, tobacco, lettuce and rice producing regenerated plants with the expected mutations and without foreign DNA (Woo et al., 2015). Such approach could be useful to eliminate pararetroviruses that are able to integrate their genomes in the host genome. One of the most important cases is the banana streak viruses (BSVs), because the cultivated banana species are only reproduced by vegetative propagation and BSVs remain inserted into genome indefinitely. Along with BSVs, *Banana bunchy top virus* (BBTV, family *Nanoviridae*) are the most economically important viruses for banana production worldwide (Rybicki, 2015; Mukwa et al., 2016). Interestingly, BBTV is a DNA virus that could be directly targeted using the same transgene-free GES approach.

Currently there is a growing worldwide debate about to how to regulate research and use of plant produced with the novel genome editing technologies (Sprink et al., 2016). In the meantime, contrasting scenarios are offered. For example, on the one hand, in the European Union, many research groups are directly concerned and waiting for the European Commission’ answer regarding the legal status of gene-edited plants and whether these plants should be regulated as GMOs (Abbott, 2015). On the other hand, in the USA, several gene-edited products have already been deregulated, including the first CRISPR-edited organism, a gene-edited mushroom (Wolt et al., 2015; Waltz, 2016). Overall, regardless of the swift progresses on genome editing technologies that are expanding our boundaries on plant virus control, other factors such as regulatory frameworks, biosafety and public perception of gene-edited organisms are also important to take into account. “At the dawn of the recombinant DNA era, the most important lesson learned was that public trust in science ultimately begins with and requires ongoing transparency and open discussion” (Baltimore et al., 2015).

## Concluding Remarks

Altogether, genome editing technologies are revolutionizing the current tools for crop improvement programs, including the antiviral arsenal of plants. Nowadays, the proof of concept of zinc finger (including AZP and ZFN), Tale and CRISPR-Cas9 technologies against plant viruses have been done for at least 14 different species, mainly in the families *Geminiviridae* and *Potyviridae*. Among these technologies, CRISPR-Cas9 has been already explored for most of the viruses tested, despite the fact that it is the more recently developed GES. This fact might be explained by the simplicity of CRISPR-Cas9 designing, which can be adopted by standard biotechnological laboratories. However, off-targets effects (possible breaks in non-targeted DNA sites) continue as a major concern for application of CRISPR-Cas9 technology. A next generation sequencing (NGS) approach can be useful to provide a compelling profile of off-target cleavage sites for a given GES (Gaj et al., 2013). Likewise, NGS approaches will be useful to gain insights on pathosystems involving CRISPR-Cas9-edited plants and targeted viruses (Hadidi et al., 2016). Beyond the debate on regulation of GES-edited plant products, the use of GESs to induce antiviral defenses raises the question on durable resistance mediated by these technologies and the generation of challenging viral isolates. Although promising, the genome editing studies aiming to generate plant virus resistance have been carried out just to evaluate resistance against specific viruses. However, further studies addressing a larger spectrum of viral isolates in a same species and longer period of viral exposition are needed to better understand the durable resistance mediated by GES and the possible mechanisms deployed by some viral isolates to overcome the induced antiviral defenses.

## Author Contributions

GR wrote the draft version of manuscript and CB reviewed the manuscript providing substantial contributions. Both authors read and approved the final version of the work.

## Conflict of Interest Statement

The authors declare that the research was conducted in the absence of any commercial or financial relationships that could be construed as a potential conflict of interest.

## References

[B1] Abbott A. (2015). Europe’s genetically edited plants stuck in legal limbo. *Nature* 528 319–320. 10.1038/528319a 26672535

[B2] Abudayyeh O. O. Gootenberg J. S. Konermann S. Joung J. Slaymaker I. M. Cox D. B. (2016). C2c2 is a single-component programmable RNA-guided RNA-targeting CRISPR effector. *Science* 353 aaf5573 10.1126/science.aaf5573 PMC5127784 27256883

[B3] Ali Z. Abulfaraj A. Idris A. Ali S. Tashkandi M. Mahfouz M. M. (2015). CRISPR/Cas9-mediated viral interference in plants. *Genome Biol.* 16:238 10.1186/s13059-015-0799-6 PMC4641396 26556628

[B4] Ali Z. Ali S. Tashkandi M. Zaidi S. S. Mahfouz M. M. (2016). CRISPR/Cas9-mediated immunity to geminiviruses: differential interference and evasion. *Sci. Rep.* 6:26912 10.1038/srep26912 PMC4881029 27225592

[B5] Anderson P. K. Cunningham A. A. Patel N. G. Morales F. J. Epstein P. R. Daszak P. (2004). Emerging infectious diseases of plants: pathogen pollution, climate change and agrotechnology drivers. *Trends Ecol. Evol.* 19 535–544. 10.1016/j.tree.2004.07.021 16701319

[B6] Baltes N. J. Hummel A. W. Konecna E. Cegan C. Bruns A. N. Bisaro D. M. (2015). Conferring resistance to geminiviruses with the CRISPR–Cas prokaryotic immune system. *Nat. Plants* 1:15145 10.1038/nplants.2015.145 PMC8612103 34824864

[B7] Baltimore D. Berg P. Botchan M. Carroll D. Charo R. Church G. (2015). Biotechnology. A prudent path forward for genomic engineering and germline gene modification. *Science* 348 36–38. 10.1126/science.aab1028 25791083 PMC4394183

[B8] Barrangou R. Fremaux C. Deveau H. Richards M. Boyaval P. Moineau S. (2007). CRISPR provides acquired resistance against viruses in prokaryotes. *Science* 315 1709–1712. 10.1126/science.1138140 17379808

[B9] Barrangou R. Marraffini L. A. (2014). CRISPR-Cas systems: prokaryotes upgrade to adaptive immunity. *Mol. Cell* 54 234–244. 10.1016/j.molcel.2014.03.011 24766887 PMC4025954

[B10] Bartel D. P. (2009). MicroRNAs: target recognition and regulatory functions. *Cell* 136 215–233. 10.1016/j.cell.2009.01.002 19167326 PMC3794896

[B11] Baulcombe D. C. (1996). Mechanisms of pathogen-derived resistance to viruses in transgenic plants. *Plant Cell* 8 1833–1844. 10.1105/tpc.8.10.1833 12239365 PMC161318

[B12] Bitinaite J. Wah D. A. Aggarwal A. K. Schildkraut I. (1998). FokI dimerization is required for DNA cleavage. *Proc. Natl. Acad. Sci. U.S.A.* 95 10570–10575. 10.1073/pnas.95.18.10570 9724744 PMC27935

[B13] Bloom K. Mussolino C. Arbuthnot P. (2015). Transcription activator-like effector (TALE) nucleases and repressor TALEs for antiviral gene therapy. *Curr. Stem Cell Rep.* 1 1–8. 10.1007/s40778-014-0008-7

[B14] Boch J. Bonas U. (2010). Xanthomonas AvrBs3 family-type III effectors: discovery and function. *Annu. Rev. Phytopathol.* 48 419–436. 10.1146/annurev-phyto-080508-081936 19400638

[B15] Boch J. Scholze H. Schornack S. Landgraf A. Hahn S. Kay S. (2009). Breaking the code of DNA binding specificity of TAL-type III effectors. *Science* 326 1509–1512. 10.1126/science.1178811 19933107

[B16] Boeke J. D. Eickbush T. Sandmeyer S. B. Voytas D. F. (2012). “Pseudoviridae,” in *Virus Taxonomy: Ninth Report of the International Committee on Taxonomy of Viruses* eds King A. M. Q. Adams M. J. Carstens E. B. Lefkowitz E. J. (Amsterdam: Elsevier/Academic Press) 467–476.

[B17] Boettcher M. McManus M. T. (2015). Choosing the right tool for the job: RNAi, TALEN, or CRISPR. *Mol. Cell* 58 575–585. 10.1016/j.molcel.2015.04.028 26000843 PMC4441801

[B18] Bragard C. Caciagli P. Lemaire O. Lopez-Moya J. J. MacFarlane S. Peters D. (2013). Status and prospects of plant virus control through interference with vector transmission. *Annu. Rev. Phytopathol.* 51 177–201. 10.1146/annurev-phyto-082712-102346 23663003

[B19] Brouns S. J. Jore M. M. Lundgren M. Westra E. R. Slijkhuis R. J. Snijders A. P. (2008). Small CRISPR RNAs guide antiviral defense in prokaryotes. *Science* 321 960–964. 10.1126/science.1159689 18703739 PMC5898235

[B20] Carroll D. (2011). Genome engineering with zinc-finger nucleases. *Genetics* 188 773–782. 10.1534/genetics.111.131433 21828278 PMC3176093

[B21] Cermak T. Doyle E. L. Christian M. Wang L. Zhang Y. Schmidt C. (2011). Efficient design and assembly of custom TALEN and other TAL effector-based constructs for DNA targeting. *Nucleic Acids Res.* 39:e82 10.1093/nar/gkr218 PMC3130291 21493687

[B22] Chandrasekaran J. Brumin M. Wolf D. Leibman D. Klap C. Pearlsman M. (2016). Development of broad virus resistance in non-transgenic cucumber using CRISPR/Cas9 technology. *Mol. Plant Pathol.* 17 1140–1153. 10.1111/mpp.12375 26808139 PMC6638350

[B23] Chen W. Qian Y. Wu X. Sun Y. Wu X. Cheng X. (2014). Inhibiting replication of begomoviruses using artificial zinc finger nucleases that target viral-conserved nucleotide motif. *Virus Genes* 48 494–501. 10.1007/s11262-014-1041-4 24474330

[B24] Cheng X. Li F. Cai J. Chen W. Zhao N. Sun Y. (2015). Artificial TALE as a convenient protein platform for engineering broad-spectrum resistance to begomoviruses. *Viruses* 7 4772–4782. 10.3390/v7082843 26308041 PMC4576204

[B25] Christian M. Cermak T. Doyle E. L. Schmidt C. Zhang F. Hummel A. (2010). Targeting DNA double-strand breaks with TAL effector nucleases. *Genetics* 186 757–761. 10.1534/genetics.110.120717 20660643 PMC2942870

[B26] Clasen B. M. Stoddard T. J. Luo S. Demorest Z. L. Li J. Cedrone F. (2016). Improving cold storage and processing traits in potato through targeted gene knockout. *Plant Biotechnol. J.* 14 169–176. 10.1111/pbi.12370 25846201 PMC11389148

[B27] D’Halluin K. Vanderstraeten C. Van Hulle J. Rosolowska J. Van Den Brande I. Pennewaert A. (2013). Targeted molecular trait stacking in cotton through targeted double-strand break induction. *Plant Biotechnol. J.* 11 933–941. 10.1111/pbi.12085 23777410 PMC4272417

[B28] Dominguez A. A. Lim W. A. Qi L. (2016). Beyond editing: repurposing CRISPR-Cas9 for precision genome regulation and interrogation. *Nat. Rev. Mol. Cell Biol.* 17 5–15. 10.1038/nrm.2015.2 26670017 PMC4922510

[B29] Ebina H. Misawa N. Kanemura Y. Koyanagi Y. (2013). Harnessing the CRISPR/Cas9 system to disrupt latent HIV-1 provirus. *Sci. Rep.* 3:2510 10.1038/srep02510 PMC3752613 23974631

[B30] Ecker J. R. Davis R. W. (1986). Inhibition of gene expression in plant cells by expression of antisense RNA. *Proc. Natl. Acad. Sci. U.S.A.* 83 5372–5376. 10.1073/pnas.83.15.5372 16593734 PMC386288

[B31] Eickbush T. Boeke J. D. Sandmeyer S. B. Voytas D. F. (2012). “Metaviridae,” in *Virus Taxonomy: Ninth Report of the International Committee on Taxonomy of Viruses* eds King A. M. Q. Adams M. J. Carstens E. B. Lefkowitz E. J. (Amsterdam: Elsevier/Academic Press) 456–466.

[B32] Estevan J. Maréna A. Callot C. Lacombe S. Moretti A. Caranta C. (2014). Specific requirement for translation initiation factor 4E or its isoform drives plant host susceptibility to Tobacco etch virus. *BMC Plant Biol.* 14:67 10.1186/1471-2229-14-67 PMC3999954 24645730

[B33] Fire A. Xu S. Montgomery M. K. Kostas S. A. Driver S. E. Mello C. C. (1998). Potent and specific genetic interference by double-stranded RNA in *Caenorhabditis elegans*. *Nature* 391 806–811. 10.1038/35888 9486653

[B34] Gaj T. Gersbach C. A. Barbas C. F. (2013). ZFN, TALEN, and CRISPR/Cas-based methods for genome engineering. *Trends Biotechnol.* 31 397–405. 10.1016/j.tibtech.2013.04.004 23664777 PMC3694601

[B35] Gelvin S. B. (2003). *Agrobacterium*-mediated plant transformation: the biology behind the “Gene-Jockeying” tool. *Microbiol. Mol. Biol. Rev.* 67 16–37. 10.1128/MMBR.67.1.16-37.2003 12626681 PMC150518

[B36] Gómez P. Rodríguez-Hernández A. M. Moury B. Aranda M. A. (2009). Genetic resistance for the sustainable control of plant virus disease: breeding, mechanisms and durability. *Eur. J. Plant Pathol.* 125 1–22. 10.1007/s10658-009-9468-5

[B37] Govindan G. Ramalingam S. (2016). Programmable site-specific nucleases for targeted genome engineering in higher eukaryotes. *J. Cell Physiol.* 231 2380–2392. 10.1002/jcp.25367 26945523

[B38] Grosse S. Huot N. Mahiet C. Arnould S. Barradeau S. Clerre D. L. (2011). Meganuclease-mediated inhibition of HSV1 infection in cultured cells. *Mol. Ther.* 19 694–702. 10.1038/mt.2010.302 21224832 PMC3070101

[B39] Hadidi A. Flores R. Candresse T. Barba M. (2016). Next-generation sequencing and genome editing in plant virology. *Front. Plant Sci.* 7:1325 10.3389/fmicb.2016.01325 PMC4999435 27617007

[B40] Hanley-Bowdoin L. Settlage S. B. Orozco B. M. Nagar S. Robertson D. (2000). Geminiviruses: models for plant DNA replication, transcription, and cell cycle regulation. *Crit. Rev. Biochem. Mol. Biol.* 35 105–140. 10821479

[B41] Hull R. (2014). *Plant Virology.* San Diego, CA: Elsevier Academic Press.

[B42] ICTV (2015). ICTV Master Species List 2015 v1. Available at: https://talk.ictvonline.org/files/master-species-lists/m/msl/5945 [accessed August 20, 2014].

[B43] Ji X. Zhang H. Zhang Y. Wang Y. Gao C. (2015). Establishing a CRISPR-Cas-like immune system conferring DNA virus resistance in plants. *Nat. Plants* 1:15144 10.1038/nplants.2015.144 27251395

[B44] Jinek M. Chylinski K. Fonfara I. Hauer M. Doudna J. A. Charpentier E. (2012). A programmable dual-RNA-guided DNA endonuclease in adaptive bacterial immunity. *Science* 337 816–821. 10.1126/science.1225829 22745249 PMC6286148

[B45] Jurica M. S. Stoddard B. L. (1999). Homing endonucleases: structure, function and evolution. *Cell Mol. Life Sci.* 55 1304–1326. 10.1007/s000180050372 10487208 PMC11146876

[B46] Kang B. C. Yeam I. Jahn M. M. (2005). Genetics of plant virus resistance. *Annu. Rev. Phytopathol.* 43 581–621. 10.1146/annurev.phyto.43.011205.141140 16078896

[B47] Kikkert J. R. Vidal J. R. Reisch B. I. (2005). Stable transformation of plant cells by particle bombardment/biolistics. *Methods Mol. Biol.* 286 61–78. 15310913 10.1385/1-59259-827-7:061

[B48] Kim H. J. Lee H. J. Kim H. Cho S. W. Kim J. S. (2009). Targeted genome editing in human cells with zinc finger nucleases constructed via modular assembly. *Genome Res.* 19 1279–1288. 10.1101/gr.089417.108 19470664 PMC2704428

[B49] Koshino-Kimura Y. Takenaka K. Domoto F. Ohashi M. Miyazaki T. Aoyama Y. (2009). Construction of plants resistant to TYLCV by using artificial zinc-finger proteins. *Nucleic Acids Symp. Ser. (Oxf.)* 53 281–282. 10.1093/nass/nrp141 19749370

[B50] Liang Z. Zhang K. Chen K. Gao C. (2014). Targeted mutagenesis in *Zea mays* using TALENs and the CRISPR/Cas system. *J. Genet. Genomics* 41 63–68. 10.1016/j.jgg.2013.12.001 24576457

[B51] Mahfouz M. M. Li L. Shamimuzzaman M. Wibowo A. Fang X. Zhu J. K. (2011). De novo-engineered transcription activator-like effector (TALE) hybrid nuclease with novel DNA binding specificity creates double-strand breaks. *Proc. Natl. Acad. Sci. U.S.A.* 108 2623–2628. 10.1073/pnas.1019533108 21262818 PMC3038751

[B52] Makarova K. S. Wolf Y. I. Alkhnbashi O. S. Costa F. Shah S. A. Saunders S. J. (2015). An updated evolutionary classification of CRISPR-Cas systems. *Nat. Rev. Microbiol.* 13 722–736. 10.1038/nrmicro3569 26411297 PMC5426118

[B53] Mandadi K. K. Scholthof K. B. (2013). Plant immune responses against viruses: How does a virus cause disease? *Plant Cell* 25 1489–1505. 10.1105/tpc.113.111658 23709626 PMC3694688

[B54] Mori T. Takenaka K. Domoto F. Aoyama Y. Sera T. (2013). Inhibition of binding of tomato yellow leaf curl virus rep to its replication origin by artificial zinc-finger protein. *Mol. Biotechnol.* 54 198–203. 10.1007/s12033-012-9552-5 22576255

[B55] Mukwa L. F. Gillis A. Vanhese V. Romay G. Galzi S. Laboureau N. (2016). Low genetic diversity of Banana bunchy top virus, with a sub-regional pattern of variation, in Democratic Republic of Congo. *Virus Genes* 52 900–905. 10.1007/s11262-016-1383-1 27550369

[B56] Nicaise V. (2014). Crop immunity against viruses: outcomes and future challenges. *Front. Plant Sci.* 5:660 10.3389/fpls.2014.00660 PMC4240047 25484888

[B57] O’Connell M. R. Oakes B. L. Sternberg S. H. East-Seletsky A. Kaplan M. Doudna J. A. (2014). Programmable RNA recognition and cleavage by CRISPR/Cas9. *Nature* 516 263–266. 10.1038/nature13769 25274302 PMC4268322

[B58] Ordiz M. I. Magnenat L. Barbas C. F. Beachy R. N. (2010). Negative regulation of the RTBV promoter by designed zinc finger proteins. *Plant Mol. Biol.* 72 621–630. 10.1007/s11103-010-9600-0 20169401

[B59] Pâques F. Duchateau P. (2007). Meganucleases and DNA double-strand break-induced recombination: perspectives for gene therapy. *Curr. Gene Ther.* 7 49–66. 10.2174/156652307779940216 17305528

[B60] Pesce C. Bolot S. Cunnac S. Portier P. Fischer-Le Saux M. Jacques M. A. (2015). High-quality draft genome sequence of the *Xanthomonas translucens* pv. cerealis pathotype strain CFBP 2541. *Genome Announc.* 3:e1574-14. 10.1128/genomeA.01574-14 PMC4333671 25676771

[B61] Piron F. Nicolaï M. Minoïa S. Piednoir E. Moretti A. Salgues A. (2010). An induced mutation in tomato eIF4E leads to immunity to two potyviruses. *PLoS ONE* 5:e11313 10.1371/journal.pone.0011313 PMC2892489 20593023

[B62] Price A. A. Grakoui A. Weiss D. S. (2016). Harnessing the prokaryotic adaptive immune system as a eukaryotic antiviral defense. *Trends Microbiol.* 24 294–306. 10.1016/j.tim.2016.01.005 26852268 PMC4808413

[B63] Price A. A. Sampson T. R. Ratner H. K. Grakoui A. Weiss D. S. (2015). Cas9-mediated targeting of viral RNA in eukaryotic cells. *Proc. Natl. Acad. Sci. U.S.A.* 112 6164–6169. 10.1073/pnas.1422340112 25918406 PMC4434742

[B64] Prins M. Laimer M. Noris E. Schubert J. Wassenegger M. Tepfer M. (2008). Strategies for antiviral resistance in transgenic plants. *Mol. Plant Pathol.* 9 73–83. 10.1111/j.1364-3703.2007.00447.x 18705886 PMC6640351

[B65] Pyott D. E. Sheehan E. Molnar A. (2016). Engineering of CRISPR/Cas9-mediated potyvirus resistance in transgene-free *Arabidopsis* plants. *Mol. Plant Pathol.* 17 1276–1288. 10.1111/mpp.12417 27103354 PMC5026172

[B66] Rizvi I. Choudhury N. R. Tuteja N. (2015). Insights into the functional characteristics of geminivirus rolling-circle replication initiator protein and its interaction with host factors affecting viral DNA replication. *Arch. Virol.* 160 375–387. 10.1007/s00705-014-2297-7 25449306

[B67] Robaglia C. Caranta C. (2006). Translation initiation factors: a weak link in plant RNA virus infection. *Trends Plant Sci.* 11 40–45. 10.1016/j.tplants.2005.11.004 16343979

[B68] Rybicki E. P. (2015). A Top Ten list for economically important plant viruses. *Arch. Virol.* 160 17–20. 10.1007/s00705-014-2295-9 25430908

[B69] Saharan V. Jain D. Pareek S. Ajay P. Kumaraswamy R. V. Kumari Jakhar S. (2016). “Viral, fungal and bacterial disease resistance in transgenic plants,” in *Advances in Plant Breeding Strategies: Agronomic, Abiotic and Biotic Stress Traits* eds Al-Khayri J. M. Jain S. M. Johnson D. V. (Cham: Springer International Publishing) 627–656.

[B70] Sampson T. R. Saroj S. D. Llewellyn A. C. Tzeng Y. L. Weiss D. S. (2013). CRISPR/Cas system mediates bacterial innate immune evasion and virulence. *Nature* 497 254–257. 10.1038/nature12048 23584588 PMC3651764

[B71] Sanfaçon H. (2015). Plant Translation Factors and Virus Resistance. *Viruses* 7 3392–3419. 10.3390/v7072778 26114476 PMC4517107

[B72] Sanford J. C. Johnston S. A. (1985). The concept of parasite-derived resistance-Deriving resistance genes from the parasite’s own genome. *J. Theor. Biol.* 113 395–405. 10.1016/S0022-5193(85)80234-4

[B73] Schornack S. Moscou M. J. Ward E. R. Horvath D. M. (2013). Engineering plant disease resistance based on TAL effectors. *Annu. Rev. Phytopathol.* 51 383–406. 10.1146/annurev-phyto-082712-102255 23725472

[B74] Sera T. (2005). Inhibition of virus DNA replication by artificial zinc finger proteins. *J. Virol.* 79 2614–2619. 10.1128/JVI.79.4.2614-2619.2005 15681461 PMC546585

[B75] Shi J. Wang H. Wu Y. Hazebroek J. Meeley R. B. Ertl D. S. (2003). The maize low-phytic acid mutant lpa2 is caused by mutation in an inositol phosphate kinase gene. *Plant Physiol.* 131 507–515. 10.1104/pp.014258 12586875 PMC166827

[B76] Shmakov S. Abudayyeh O. O. Makarova K. S. Wolf Y. I. Gootenberg J. S. Semenova E. (2015). Discovery and functional characterization of diverse class 2 CRISPR-cas systems. *Mol. Cell* 60 385–397. 10.1016/j.molcel.2015.10.008 26593719 PMC4660269

[B77] Silva G. Poirot L. Galetto R. Smith J. Montoya G. Duchateau P. (2011). Meganucleases and other tools for targeted genome engineering: perspectives and challenges for gene therapy. *Curr. Gene Ther.* 11 11–27. 10.2174/156652311794520111 21182466 PMC3267165

[B78] Simón-Mateo C. García J. A. (2011). Antiviral strategies in plants based on RNA silencing. *Biochim. Biophys. Acta* 1809 722–731. 10.1016/j.bbagrm.2011.05.011 21652000

[B79] Sprink T. Eriksson D. Schiemann J. Hartung F. (2016). Regulatory hurdles for genome editing: process- vs. product-based approaches in different regulatory contexts. *Plant Cell Rep.* 35 1493–1506. 10.1007/s00299-016-1990-2 27142995 PMC4903111

[B80] Steinert J. Schiml S. Puchta H. (2016). Homology-based double-strand break-induced genome engineering in plants. *Plant Cell Rep.* 35 1429–1438. 10.1007/s00299-016-1981-3 27084537

[B81] Sternberg S. H. Redding S. Jinek M. Greene E. C. Doudna J. A. (2014). DNA interrogation by the CRISPR RNA-guided endonuclease Cas9. *Nature* 507 62–67. 10.1038/nature13011 24476820 PMC4106473

[B82] Szittya G. Burgyán J. (2013). RNA interference-mediated intrinsic antiviral immunity in plants. *Curr. Top. Microbiol. Immunol.* 371 153–181. 10.1007/978-3-642-37765-5_6 23686235

[B83] Takenaka K. Koshino-Kimura Y. Aoyama Y. Sera T. (2007). Inhibition of tomato yellow leaf curl virus replication by artificial zinc-finger proteins. *Nucleic Acids Symp. Ser. (Oxf.)* 51 429–430. 10.1093/nass/nrm215 18029770

[B84] Tebas P. Stein D. Tang W. W. Frank I. Wang S. Q. Lee G. (2014). Gene editing of CCR5 in autologous CD4 T cells of persons infected with HIV. *N. Engl. J. Med.* 370 901–910. 10.1056/NEJMoa1300662 24597865 PMC4084652

[B85] Townsend J. A. Wright D. A. Winfrey R. J. Fu F. Maeder M. L. Joung J. K. (2009). High-frequency modification of plant genes using engineered zinc-finger nucleases. *Nature* 459 442–445. 10.1038/nature07845 19404258 PMC2743854

[B86] Urnov F. Rebar E. J. Holmes M. C. Zhang H. S. Gregory P. D. (2010). Genome editing with engineered zinc finger nucleases. *Nat. Rev. Genet.* 11 636–646. 10.1038/nrg2842 20717154

[B87] Voytas D. F. (2013). Plant genome engineering with sequence-specific nucleases. *Annu. Rev. Plant Biol.* 64 327–350. 10.1146/annurev-arplant-042811-105552 23451779

[B88] Waltz E. (2016). Gene-edited CRISPR mushroom escapes US regulation. *Nature* 532:293 10.1038/nature.2016.19754 27111611

[B89] Wang Y. Cheng X. Shan Q. Zhang Y. Liu J. Gao C. (2014). Simultaneous editing of three homoeoalleles in hexaploid bread wheat confers heritable resistance to powdery mildew. *Nat. Biotechnol.* 32 947–951. 10.1038/nbt.2969 25038773

[B90] Wani S. Sanghera G. S. (2010). Genetic engineering for viral disease management in plants. *Not. Sci. Biol.* 2 20–28. 10.15835/nsb.2.1.3614

[B91] Wilson R. C. Doudna J. A. (2013). Molecular mechanisms of RNA interference. *Annu. Rev. Biophys.* 42 217–239. 10.1146/annurev-biophys-083012-130404 23654304 PMC5895182

[B92] Wolt J. D. Wang K. Yang B. (2015). The regulatory status of genome-edited crops. *Plant Biotechnol. J.* 14 510–518. 10.1111/pbi.12444 26251102 PMC5042095

[B93] Woo J. W. Kim J. Kwon S. I. Corvalán C. Cho S. W. Kim H. (2015). DNA-free genome editing in plants with preassembled CRISPR-Cas9 ribonucleoproteins. *Nat. Biotechnol.* 33 1162–1164. 10.1038/nbt.3389 26479191

[B94] Wyman C. Kanaar R. (2006). DNA double-strand break repair: all’s well that ends well. *Annu. Rev. Genet.* 40 363–383. 10.1146/annurev.genet.40.110405.090451 16895466

[B95] Xie K. Minkenberg B. Yang Y. (2015). Boosting CRISPR/Cas9 multiplex editing capability with the endogenous tRNA-processing system. *Proc. Natl. Acad. Sci. U.S.A.* 112 3570–3575. 10.1073/pnas.1420294112 25733849 PMC4371917

[B96] Xing H. L. Dong L. Wang Z. P. Zhang H. Y. Han C. Y. Liu B. (2014). A CRISPR/Cas9 toolkit for multiplex genome editing in plants. *BMC Plant Biol.* 14:327 10.1186/s12870-014-0327-y PMC4262988 25432517

[B97] Ye X. (2015). Development and application of plant transformation techniques. *J. Integr. Agric.* 14 411–413. 10.1016/S2095-3119(14)60945-X

[B98] Younis A. Siddique M. I. Kim C. K. Lim K. B. (2014). RNA interference (RNAi) induced gene silencing: a promising approach of hi-tech plant breeding. *Int. J. Biol. Sci.* 10 1150–1158. 10.7150/ijbs.10452 25332689 PMC4202031

[B99] Ziebell H. (2016). “Plant defence and viral interference,” in *Plant-Virus Interactions* ed. Kleinow T. (Cham: Springer International Publishing) 123–159.

